# Early and late effects of volatile sedation with sevoflurane on respiratory mechanics of critically ill COPD patients

**DOI:** 10.1186/s13613-024-01311-4

**Published:** 2024-06-18

**Authors:** Boris Jung, Maxime Fosset, Matthieu Amalric, Elias Baedorf-Kassis, Brian O’Gara, Todd Sarge, Valerie Moulaire, Vincent Brunot, Arnaud Bourdin, Nicolas Molinari, Stefan Matecki

**Affiliations:** 1 Medical Intensive Care Unit, Montpellier University and Montpellier University Health Care Center, Montpellier, 34295 France; 2 PhyMedExp laboratory, Montpellier University, INSERM, CNRS, CHRU Montpellier, Montpellier, 34295 France; 3 IMAG, CNRS, Montpellier University and Montpellier University Health Care Center, Montpellier, 34295 France; 4 Department of Anesthesia, Critical Care and Pain Medicine, Beth Israel Deaconess Medical Center Harvard Medical School, Boston, MA USA; 5 Division of Pulmonary, Sleep and Critical Care Medicine, Beth Israel Deaconess Medical Center, Harvard Medical School Boston, Boston, MA USA; 6 Department of Respiratory Diseases, Montpellier University and Montpellier University Health Care Center, Montpellier, 34295 France

**Keywords:** COPD, Volatile sedation, Sevoflurane, Mechanical ventilation, Respiratory mechanics

## Abstract

**Background:**

The objective was to compare sevoflurane, a volatile sedation agent with potential bronchodilatory properties, with propofol on respiratory mechanics in critically ill patients with COPD exacerbation.

**Methods:**

Prospective study in an ICU enrolling critically ill intubated patients with severe COPD exacerbation and comparing propofol and sevoflurane after 1:1 randomisation. Respiratory system mechanics (airway resistance, PEEPi, trapped volume, ventilatory ratio and respiratory system compliance), gas exchange, vitals, safety and outcome were measured at inclusion and then until H48. Total airway resistance change from baseline to H48 in both sevoflurane and propofol groups was the main endpoint.

**Results:**

Sixteen patients were enrolled and were sedated for 126 h(61–228) in the propofol group and 207 h(171–216) in the sevoflurane group. At baseline, airway resistance was 21.6cmH2O/l/s(19.8–21.6) in the propofol group and 20.4cmH2O/l/s(18.6–26.4) in the sevoflurane group, (*p* = 0.73); trapped volume was 260 ml(176–290) in the propofol group and 73 ml(35–126) in the sevoflurane group, *p* = 0.02. Intrinsic PEEP was 1.5cmH2O(1–3) in both groups after external PEEP optimization. There was neither early (H4) or late (H48) significant difference in airway resistance and respiratory mechanics parameters between the two groups.

**Conclusions:**

In critically ill patients intubated with COPD exacerbation, there was no significant difference in respiratory mechanics between sevoflurane and propofol from inclusion to H4 and H48.

**Supplementary Information:**

The online version contains supplementary material available at 10.1186/s13613-024-01311-4.

## Background

Patients developing an acute exacerbation of Chronic Obstructive Pulmonary Disease (COPD) demonstrate a sudden worsening of respiratory symptoms and expiratory flow limitation [1]. Expiratory flow limitation develops secondary to increased airway resistance from mucosal edema, mucus production with impaired clearance and bronchospasm [2, 3]. Flow limitation may result in dynamic hyperinflation and breath stacking due to insufficient expiratory time to allow patients to return to the relaxation volume prior to the subsequent breath. This combination of flow limitation and dynamic hyperinflation may lead to increased work of breathing and respiratory failure that requires invasive mechanical support, while providing sufficient time for bronchodilators, steroids and treatment of any additional infection to improve the flow limitation [1, 4]. During mechanical ventilation, patients with COPD exacerbations typically require sedatives to allow for tolerance of potentially uncomfortable settings. As such, the use of sedative agents which may further augment bronchodilation is of significant interest to investigate [5].

Sevoflurane and isoflurane are volatile sedative agents with which have both demonstrated bronchodilatory properties in a similar extent [6–8]. They can be routinely administered in the Intensive Care Unit (ICU) using miniature vaporizers or reflection devices [9]. These agents have been successfully used in refractory status asthmaticus with multiple case series’ describing a rapid decrease in airway resistance, dynamic hyperinflation, and accelerated carbon dioxide clearance [10]. However, no study has yet evaluated the use of volatile sedation in critically ill patients with COPD exacerbation.

We have designed the SEVOCOPD study to evaluate the respiratory mechanics over time in critically ill patients with COPD receiving invasive mechanical ventilation (MV) and sedation with either intravenous propofol or the volatile anesthetic sevoflurane.

We hypothesized that volatile sedation would result in a further early and late decrease in airway resistance compared to intravenous sedation with propofol.

## Methods

### Study setting and design

We performed a single center open-label study in a tertiary teaching medical center. The study protocol and statistical analysis plan was approved by a central ethics committee (Comité de Protection des Personnes Sud Méditerranée, Nice, France, 2017002504-27) in accordance with both French law and the Declaration of Helsinki. We obtained written informed consent from the patient or a relative upon study inclusion. However, considering the severity of the illness, the central ethics committee allowed the investigators to collect a proxy consent with a subsequent written permission to pursue the research obtained from the patient. The French National Agency for Safety in HealthCare (ANSM) oversaw the research protocol and the potential safety issues. An independent steering committee oversaw the research protocol and performed a preplanned safety analysis after half of the patients were enrolled. The protocol was registered (ClinicalTrial.gov NCT03460015, submitted 2018-03-02).

### Population

Consecutive critically ill patients with a known or suspected COPD exacerbation according to the GOLD international guidelines [1] requiring invasive MV with an expected duration of at least 24 h were considered eligible. Exclusion criteria were a contraindication to sevoflurane (personal or familial history of malignant hyperthermia, allergy to volatile sedation, uncontrolled intracranial hypertension), contraindication to propofol (allergy to propofol or soy beans, personal history of propofol related infusion syndrome). Randomization was performed using a computer-generated allocation sequence after intubation and patients were randomized to receive either sevoflurane or propofol sedation in a 1:1 allocation.

### Intervention

During the period of screening and pending consent obtainment, patients were sedated with propofol and sufentanil and active heating was performed (F850, Fisher Paykel, Auckland, NZ).

Although the present study is a physiological study, we also seek to design a study that would be safe and reproducible in larger comparative studies. After randomization, patients in the propofol group received a 2% infusion of propofol targeting a Richmond Agitation Sedation Scale (RASS) of -5 (e-Fig. 1). In the sevoflurane group, we targeted an expiratory fraction of sevoflurane of 1-1.5% to balance deep sedation and potential bronchodilation with the risk of hypotension in these critically ill patients [8, 10, 11]. Sevoflurane was administered through the SEDACONDA-ACD-S device (Sedana Medical, Danderyd, Sweden) positioned between the Y-piece and the endotracheal tube which enables the administration of volatile agents on most of the modern ICU ventilators. The SEDACONDA-ACD-S has a dead space of 50 ml, an internal resistance to airflow of 3.5cmH2O/l/s, is a heat and moisture exchanger which acts as an electrostatic bacterial/viral filter that reflects back approximately 90% of the exhaled sevoflurane to prevent from waste and pollution [12]. The additional work of breathing related to its physical characteristics is balanced with low dose of volatile sedation [13]. Sidestream CO_2_ and sevoflurane inspiratory and expiratory fractions were continuously monitored using the ICU monitor gas analyzer module (Carescape B650, GE, Boston, MA).

In both groups, pain control was assured using continuous sufentanil infusion targeting a behavioral pain scale (BPS) of 3.

A daily morning sedation interruption protocol was initiated in eligible patients after the following items were checked: absence of drug-induced paralysis, PaO2/FiO2 ratio ≥ 150 mmHg with a fraction of inspired oxygen ≤ 50% and a positive end-expiratory pressure ≤ 8 cmH20, hemodynamic stability, reason for intubation resolved, and absence of intracranial hypertension.

### Mechanical ventilation and COPD exacerbation treatments

Patients in both groups were ventilated using a Drager V600 ventilator (Lübeck, Germany). MV was set as suggested by Marini [14] assist controlled ventilation mode, tidal volume of 6–8 ml/kg ideal body weight, respiratory rate of 12–18 cycles/min, inspiratory flow of 70 L/min with square flow pattern, inspiratory/expiratory time ratio of 1/2 to 1/3, inspiratory fraction of oxygen to target a SpO2 of 88–94%. In mechanically ventilated patients with COPD decompensation, external PEEP may be associated with no change, increase or decrease (“paradoxical response”) in dynamic hyperinflation according to the patients profile (pure expiratory flow limitation with or without heterogenous lungs).

Although, approximately one third of mechanically ventilated patients with intrinsic PEEP will absorb external PEEP while others may experience hyperinflation [15], we uptitrated the external PEEP while avoiding dynamic hyperinflation by monitoring plateau pressure [14, 16].

After 48 h, the ventilator was switched to pressure support ventilation if the patient tolerated it and weaning was started according to the international guidelines [17]. Non-invasive ventilation was recommended in all patients in the post extubation period [18].

In addition to specific treatments targeting the inciting event for the COPD exacerbation, according to the GOLD guidelines [1] : all patients received a short acting beta agonist agent (albuterol, metered-dose inhaler, 200mcg, q6) and a short acting antimuscarinic agent (ipratropium, metered-dose inhaler, 20mcg, q8) using an inhalation chamber (Spirale DDS, Haylard Medical, Paris, France) placed between the endotracheal tube and the SEDACONDA-ACD-S. Every patient also received 60 mg intravenous methylprednisolone q24 for a duration of 7 days unless contra-indicated by the physician in charge. The protocol stated that cisatracurium infusion could be considered only if respiratory acidemia was severe (pH < 7.20) despite MV optimization. Other medications (helium, ketamine, magnesium, intravenous beta agonist) were not recommended.

### Data collection

In both groups, vitals, BPS and RASS, bispectral index, sufentanil and propofol dosing or sevoflurane expiratory fraction, respiratory system mechanics (maximal pressure, plateau pressure measured after a two seconds end-inspiratory hold, P1 which is the first measured pressure when inspiratory flow is equal to zero, total and intrinsic PEEP, trapped volume) and arterial blood gases were collected at early and late time points: 30 min, H4, H8, H12, H24, H36 and H48 (e-Fig. 1).

Total airway resistance was measured using the rapid interruption of inspiratory flow at the airways while measuring the airway pressure downstream the location of occlusion [19]. After occlusion the sudden pressure drop from maximal pressure to pressure at first zero flow (P1) is the pure resistive pressure drop. The slow decay from P1 to the plateau pressure is the pressure dissipation into the viscoelastic units. Then, total resistance of the respiratory system is (maximal pressure – plateau pressure)/flow, interrupter resistance is (maximal pressure - P1)/flow and additional viscoelastic resistance is total resistance – interrupter resistance. Using the data gathered from pressure sensors and flow measurements, the ventilator’s software used in the present study calculated the volume of air that remains trapped (trapped volume) and the total PEEP within the circuit or the patient’s airways at the end of expiration.

### Outcomes

The primary outcome was the late (from baseline to H48), total airway resistance variation between the two groups.

The secondary outcomes were airway resistance (early variation, from inclusion to H4), respiratory mechanics (peak pressure, trapped volume, intrinsic and total PEEP, ventilatory ratio and respiratory system compliance) variation. Arterial blood gases, duration of mechanical ventilation and survival are also presented although the study was not designed to show a difference between groups.

### Statistical analysis

No previous study has compared propofol with sevoflurane in critically ill mechanically ventilated patients with COPD exacerbation. We extrapolated the literature that has described the effect of volatile sedation in stable COPD patients in the OR [8], has described respiratory mechanics in patients with stable vs. exacerbated COPD [20] as well as the acute effect of bronchodilator in COPD patients [21]. We then extrapolated that a total of 22 patients would be needed to anticipate a difference of 7+/-5 cmH2O/l/s in total airway resistance from inclusion to H48 between the two groups. We determined this sample size taking into account a two side alpha risk of 0.05 and a statistical power of 80%. We assumed that less than 10% of patients would be non-analyzable (loss to follow up or consent withdrawal). The study was planned with a safety and utility interim analysis after half of the inclusions.

Baseline characteristics in both groups were analyzed as frequencies and percentages for categorical variables and as means and SDs or medians and IQRs and compared with parametric or non-parametric tests as appropriate.

We compared mean airway resistance and respiratory system mechanics among each group between baseline and 48 h and between baseline and 4 h as a secondary analysis. We did all the analyses with R (version 4.1.3).

## Results

### Population

**Fig. 1:**
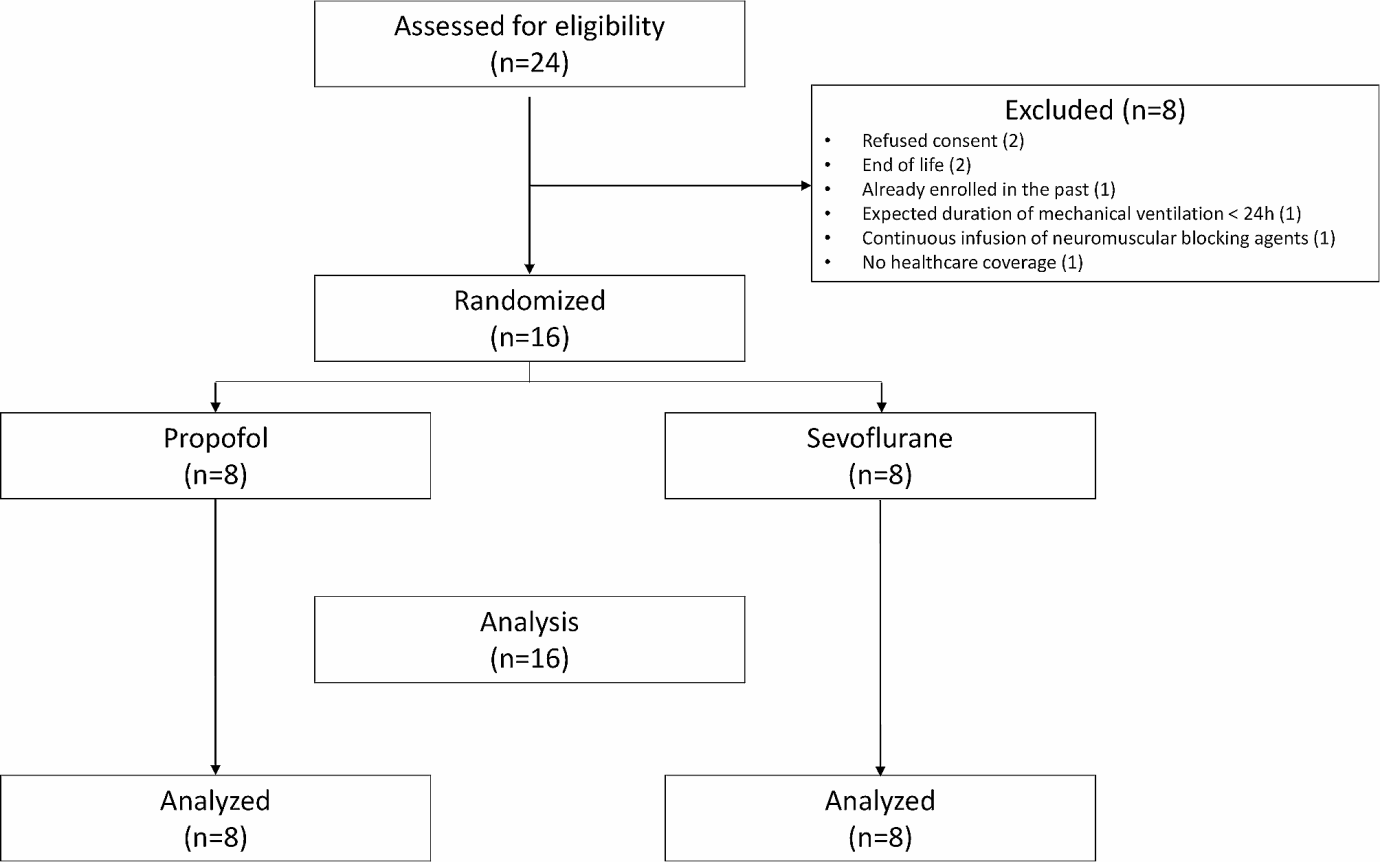
Study flow chart

The study occurred from March 1 2018 to September 19 2020 and was prematurely terminated after enrolling 16 patients out of the 22 initially planned, due to a slower enrollment rate caused by the COVID19 pandemic (Fig. 1). Table 1 present the demographic characteristics and ventilatory settings of the enrolled patients. Of the 16 patients, 13 (81%) were male, with a median age of 67 years (63–73) and a median body mass index of 24 kg/m^2^ (20–28). Five patients received long-term home oxygen therapy (Table 1). All patients were initially ventilated in assist control ventilation mode with no spontaneous breathing cycles, with a tidal volume of 7 ml/kg/IBW (6.2–7.6), respiratory rate of 18 c/min (16–18), external PEEP of 7.5 cmH2O (5–9), and FiO2 of 33% (30–36), with no significant difference between the two groups (Table 1). At baseline, median peak pressure was 42cmH2O (38–50), P1 was 21cmH2O (17–24), and plateau pressure was 18cmH2O (13–30) with no significant difference between the groups (Table 1). At baseline, trapped volume was 260 ml (176–290) in the propofol group and 73 ml (35–126) in the SEVO group (*p* = 0.02). After external PEEP optimization, PEEPi was 1.5cmH2O (1–3) in both groups. Arterial blood gases were collected upon enrollment and at each time point. At baseline, pH was 7.33 (7.30–7.37), PaO2 was 73mmHg (64–90), PaCO2 was 48mmHg (45–53), and PaO2/FiO2 was 237mmHg (226–269) (Table 1).

**Table 1:** Demographics upon admission, ventilatory settings, cardiovascular parameters, sedation and arterial blood gases at steady state after enrolment

	Propofol group (*n* = 8)	Sevoflurane group (*n* = 8)	*p*-value
Sex (male)	7 (88)	6 (75)	1
Age (yr)	66 ± 8	68 ± 6	0.62
Body Mass Index (kg/m2)	23.5 ± 3.7	25 ± 6.2	0.56
SAPSII score upon admission	44.2 ± 18.2	52.5 ± 12.5	0.31
Main cause of COPD exacerbation being lower respiratory tract infection or pneumonia	7 (88)	6 (75)	1
Long term oxygen therapy	5 (63)	6 (75)	1
Stage III or IV NYHA	6 (75)	5 (63)	1
Charlson Comorbidity Index	7 (3)	11 (7)	0.14
**Ventilatory settings upon inclusion**			
Time from intubation to enrolment	29.5 ± 28.4	15.7 ± 12.4	0.72
FiO2 (%)	31 ± 7	36 ± 7	0.28
Tidal volume (ml/kg PBW)	483 ± 63	473 ± 81	0.79
Respiratory Rate (c/min)	18 ± 0.7	17 ± 1.3	0.23
Maximal Pressure (cmH2O)	43 ± 5	44 ± 9	0.84
Plateau Pressure (cmH2O)	18 ± 4	18 ± 5	0.70
PEEPe (cmH2O)	8 ± 2	7 ± 2	0.26
PEEPi (cmH2O)	2 ± 2	2 ± 3	0.8
Total Airway Resistance (cmH2O/l/s)	22 ± 1.9	22 ± 6.8	0.86
Trapped volume (ml)	236 ± 80	108 ± 124	0.02
**Cardiovascular, sedation and arterial blood gases at steady state after enrolment**			
Mean Arterial Pressure (mmHg)	69 ± 7	76 ± 10	0.12
Heart rate (bpm)	104 ± 28	96 ± 18	1
Norepinephrine (mg/h)	1.25 ± 0.88	1.48 ± 1.18	0.67
Propofol (mg/kg/h)	2.95 ± 1.11	NA	NA
Sevoflurane (Expiratory fraction, %)	NA	1.33 ± 0.18	NA
Sufentanil (µg/h)	14 ± 4	12 ± 5	0.8
pH	7.35 ± 0.07	7.34 ± 0.11	0.46
PaCO2 (mmHg)	49 ± 7	49 ± 7	0.8
PaO2 (mmHg)	76 ± 23	86 ± 32	0.52
Bicarbonate (mmol/l)	27 ± 4	27 ± 7	0.43

NA: not applicable. NYHA: New York Heart Association, PBW: predicted body weight. Data are presented as mean and standard deviation or number and percentage

### Compliance with the study protocol

No protocol violations were observed in our study. The median RASS sedation score during the first 48 h of the protocol was − 5 (-5 to -5), and the BIS was 45 (39–52), with no statistically significant difference between the two groups. The expiratory sevoflurane fraction was 1.3% (1.1–1.5) in the sevoflurane group, and the propofol dose was 3.4 mg/kg/h (2.6–3.8) in the propofol group. Sufentanil was administered at 15mcg/h (10–15) during the first 48 h in both groups. As per the study protocol, all patients were treated with inhaled short-acting beta-agonist and antimuscarinic agents. Methylprednisolone 60 mg was administered daily to all patients except for patient n°5 per caring physician’s preference. Neuromuscular blocking agents, ketamine, magnesium sulfate, or intravenous short-acting beta-agonist agents was not used in any patient.

### Primary outcome: total airway resistance change from inclusion to H48

**Fig. 2:**
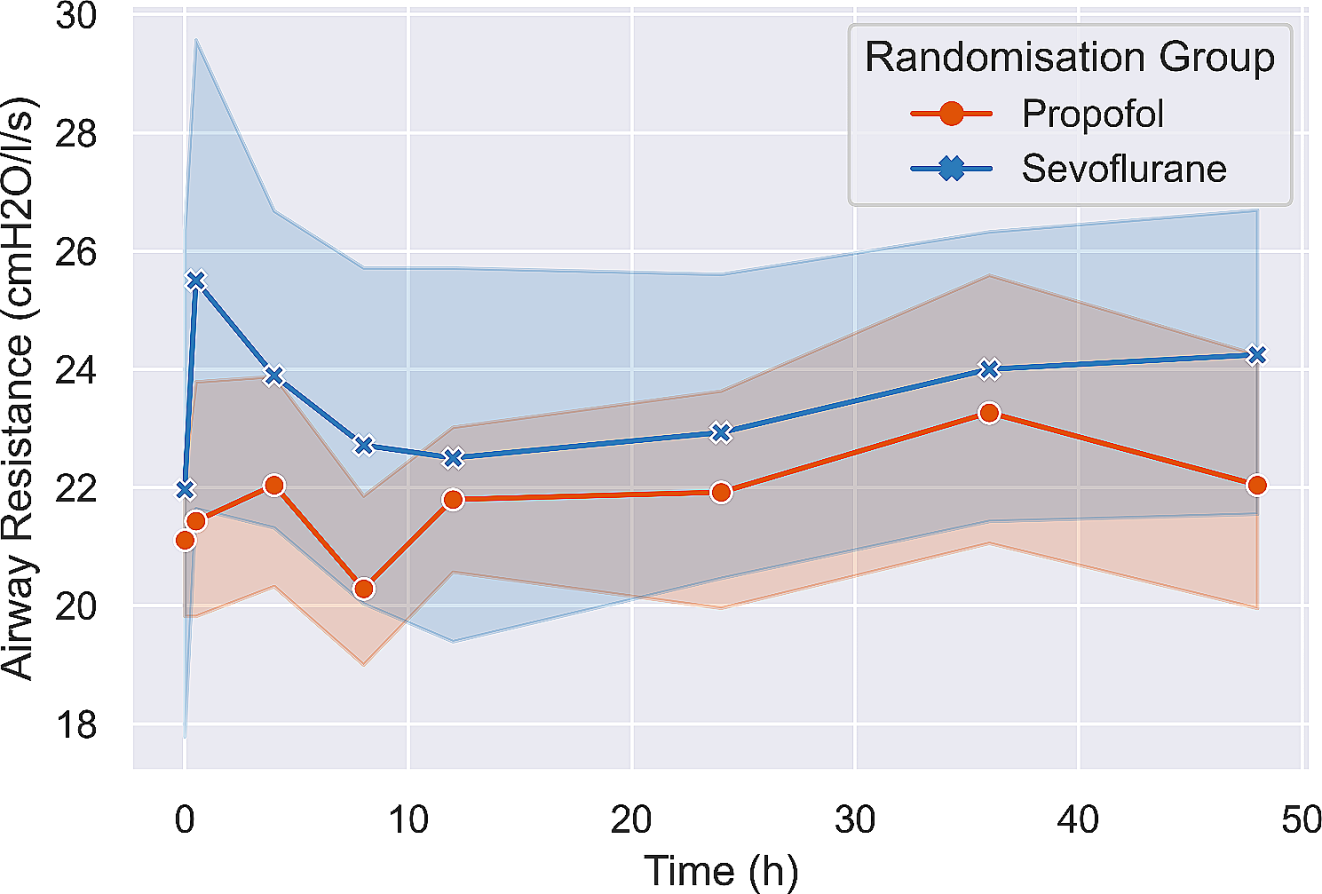
Lineplot displaying total airway resistance from enrolment to H48 in both study groups (mean +/- SEM). There was no difference in airway resistance trend among time between the two groups

Figure 2 displays the total airway resistance (mean +/- SEM) from baseline to H48. Upon inclusion, total airway resistance was 21.6cmH2O/l/s (19.8–21.6) in the propofol group and 20.4cmH2O/l/s (18.6–26.4) in the sevoflurane group, *p* = 0.73. At H48, total airway resistance was 20.4cmH2O/l/s (18.6–24.6) in the propofol group and 24.6cmH2O/l/s (21.6–27.6) in the SEVO group, *p* = 0.25. The mean total airway resistance difference between baseline and H48 in the propofol group was − 0.63cmH2O/l/s (-2.34 to 1.08) and − 1.80cmH2O/l/s (-4.83 to 1.23) in the sevoflurane group.

*Secondary outcomes: early changes in airway resistance and respiratory* mechanics.

Early change in airway resistance (from baseline to H4) is displayed on Fig. 2. There was not significant early change in airway resistance between the two groups.

**Fig. 3:**
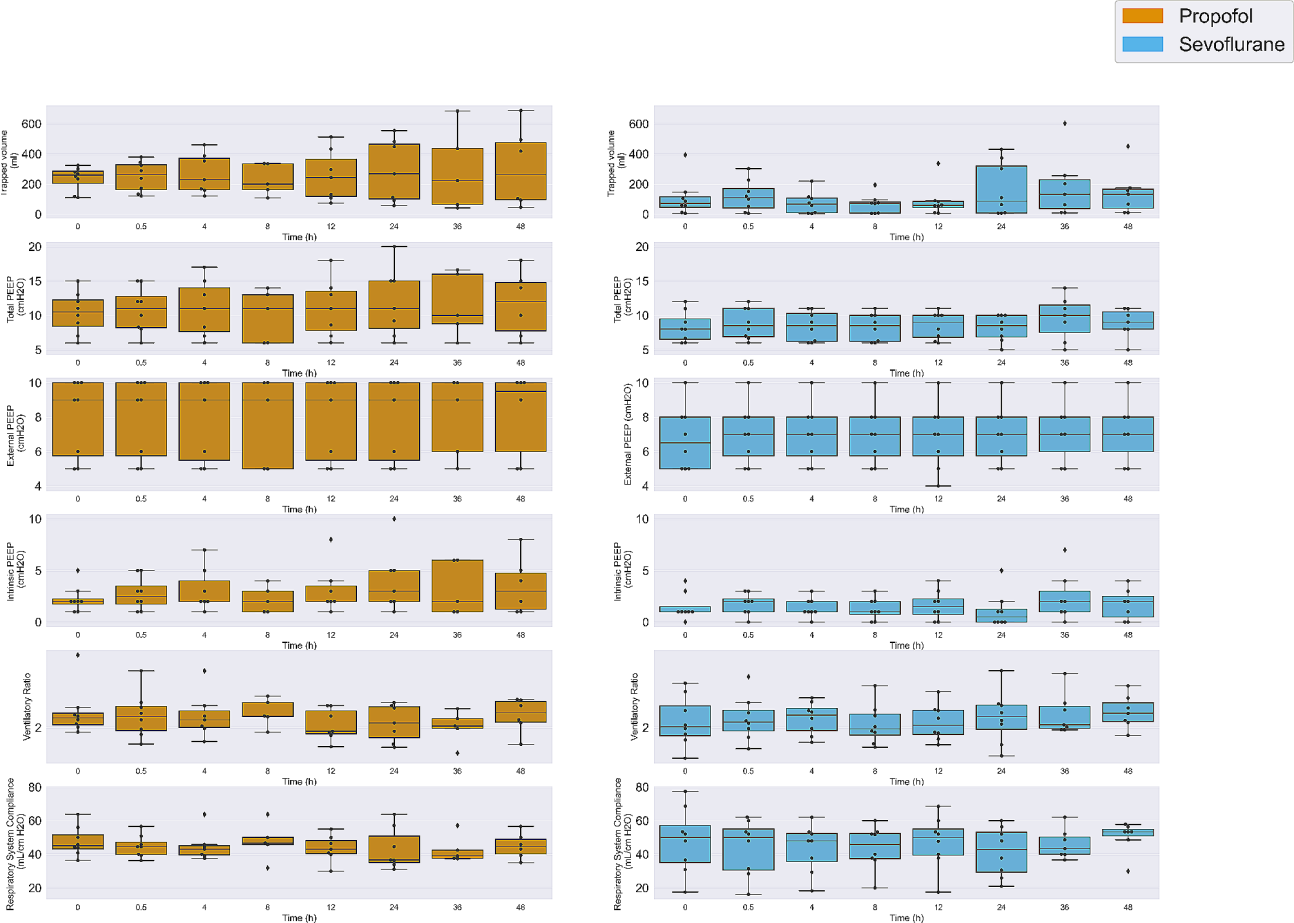
Respiratory system mechanics over time between the two groups. There was no difference in airway resistance trend among time between the two groups

Over time, there were no significant difference in hemodynamic parameters (e-Fig. 3), minute ventilation, oxygenation, or PaCO2 between the two groups (e-Fig. 2) and intrinsic PEEP, ventilatory ratio, respiratory system compliance or trapped volume (Fig. 3).

The duration of invasive mechanical ventilation was 12 days (5–20) in the propofol group and 15 days (9–21) in the sevoflurane group (*p* = 0.93). Thirteen out of sixteen patients were alive at day 90.

### Treatments side effects

The duration of sevoflurane administration was 207 h (171–216), with its maximum duration of administration in a single patient being 293 h, while the duration of propofol administration was 126 h (61–228), with its maximum duration of administration being 496 h. There was no significant difference in sedation duration between the two groups (*p* = 0.63).

We collected treatment side effects as requested by the French National Agency for Safety in Healthcare (ANSM). We reported one episode of nephrogenic diabetes insipidus in the sevoflurane group (patient n°3). The administration of sevoflurane for this patient was interrupted after 216 h, and subsequently nephrogenic diabetes insipidus was reversible.

No episode of acute exacerbated hypercapnia or severe cardiovascular events related to the study drugs was observed during the study period. Trends in arterial blood pressure, heart rate, and norepinephrine dosage in both groups are represented on e-Fig. 3.

## Discussion

In the present study we report that early and late airway resistance, peak pressure, PEEPi and trapped volume was not statistically different between patients randomized to either sedation with sevoflurane or propofol.

Isoflurane and sevoflurane are modern volatile agents approved for sedation and considered candidates for widespread use in the ICU. Sevoflurane provides rapid onset sedation and rapid clearance mostly by the lungs with no accumulation in patients with kidney or liver dysfunction, amnesia, akinesia and autonomic blockage with no concerns for tachyphylaxis [6, 12]. Although it has been associated with increased mechanical power in a bench study [22], it has been associated with decreased lung inflammation, less epithelial injury [23, 24] and improved oxygenation in patients with Acute Respiratory Distress Syndrome [25]. Long-term administration of volatile agents is non-inferior to propofol and safe [9] and we administered sevoflurane continuously for 207 h (171–216) with no severe adverse event. Compared with intravenous sedation agents, volatile agents have been associated with more rapid arousal and shorter time to extubation in the ICU [26, 27].

Volatile agents may have bronchodilatory effects that work directly by relaxing airway smooth muscle cells and indirectly by depressing protective airway reflexes [12, 28]. The use of volatile sedation has been sparsely reported in cases of refractory status asthmaticus, resulting in a rapid decrease in airway resistance, dynamic hyperinflation, and improved clearance of carbon dioxide. Although data have shown a 20 to 30% decrease in airway resistance with sevoflurane in comparison with thiopental in stable patients with COPD scheduled for surgery [8], there is no data available in critically ill patients intubated with COPD exacerbation.

Critically ill patients with COPD exacerbations who require endotracheal intubation present with dynamic hyperinflation, which may result from numerous factors, including reduced lung recoil pressure, small airway collapse, airway inflammation, mucus overproduction and reduced clearance, and varying degrees of bronchospasm [2, 3, 29]. Volatile sedation may affect gas redistribution and alveolar rate constants via bronchodilatation that could ease gas trapping. Although the pathophysiology of COPD exacerbation is complex and heterogenous among patients [2, 30], short acting bronchodilatory agents have been shown to decrease airway resistance in passive mechanically ventilated COPD patients [31]. Our study is the first conducted in the ICU and shows that sevoflurane did not decrease early or late airway resistance over time in comparison with propofol. Although not categorized as a bronchodilator agent, propofol has been reported to induce bronchodilation in patients with COPD [32] and is currently recommended as the first-line sedative drug in the ICU to avoid benzodiazepine use [33]. We observed a brief, reversible spike in airway resistance 30 min after randomisation in the sevoflurane group (Fig. 2), likely due to the internal resistance of the SEDACONDA-S device [13], which was eventually resolved after initiating sevoflurane.

The present study presents several limitations. Firstly, it was a pilot single-center physiological randomised study with a small sample size. To overcome the small pre-planned sampling size, the study protocol standardized the medical care and all patients received a similar treatment for COPD exacerbation except for the sedation with no difference between the two groups. In particular, we opted for a short-term, low-dose, systemic steroid treatment for every patient [4, 34–36]. Secondly, we did not report any inflammation or biological data and only focused on pragmatic physiological endpoints that are easily available on every ICU ventilator. Thirdly, we chose to individualize the PEEP setting according to Marini et al. [14] and according to the patients baseline respiratory mechanics rather than setting a similar PEEP for every patient [16, 29]. We then titrated the external PEEP in order to minimize dynamic hyperinflation as estimated by the plateau pressure as well as we carefully monitored the cardiovascular response to PEEP titration. Finally, although bronchodilation has been reported with higher but also lower dose of sevoflurane, the dose/bronchodilation effect is controversial [8, 10, 37]. We therefore opted for a pragmatic approach that balances the need for deep sedation typically achieved with an expiratory fraction from 0.8 to 1% in the critically ill patients [38], the potential for bronchodilation and the high risk of volatile sedation-induced hypotension in this critically ill population.

## Conclusion

In the current investigation, sevoflurane-based volatile sedation was compared with intravenous propofol in critically ill patients with COPD exacerbation and in need of invasive mechanical ventilation. Neither early nor late reductions in airway resistance were observed with either sedation method. Despite the limited power due to the premature termination of the study, no statistical evidence was found supporting any differences between the groups.

### Electronic supplementary material

Below is the link to the electronic supplementary material.

Supplementary Material 1

Supplementary Material 2

Supplementary Material 3

Supplementary Material 4

## Data Availability

The datasets used and/or analysed during the current study are available from the corresponding author on reasonable request.
