# Supplementary figures and images for: Early and late effects of volatile sedation with sevoflurane on respiratory mechanics of critically ill COPD patients
Source: Ann Intensive Care. 2024 Jun 18;14:91. doi: 10.1186/s13613-024-01311-4 (PMC11189368; doi:10.1186/s13613-024-01311-4)

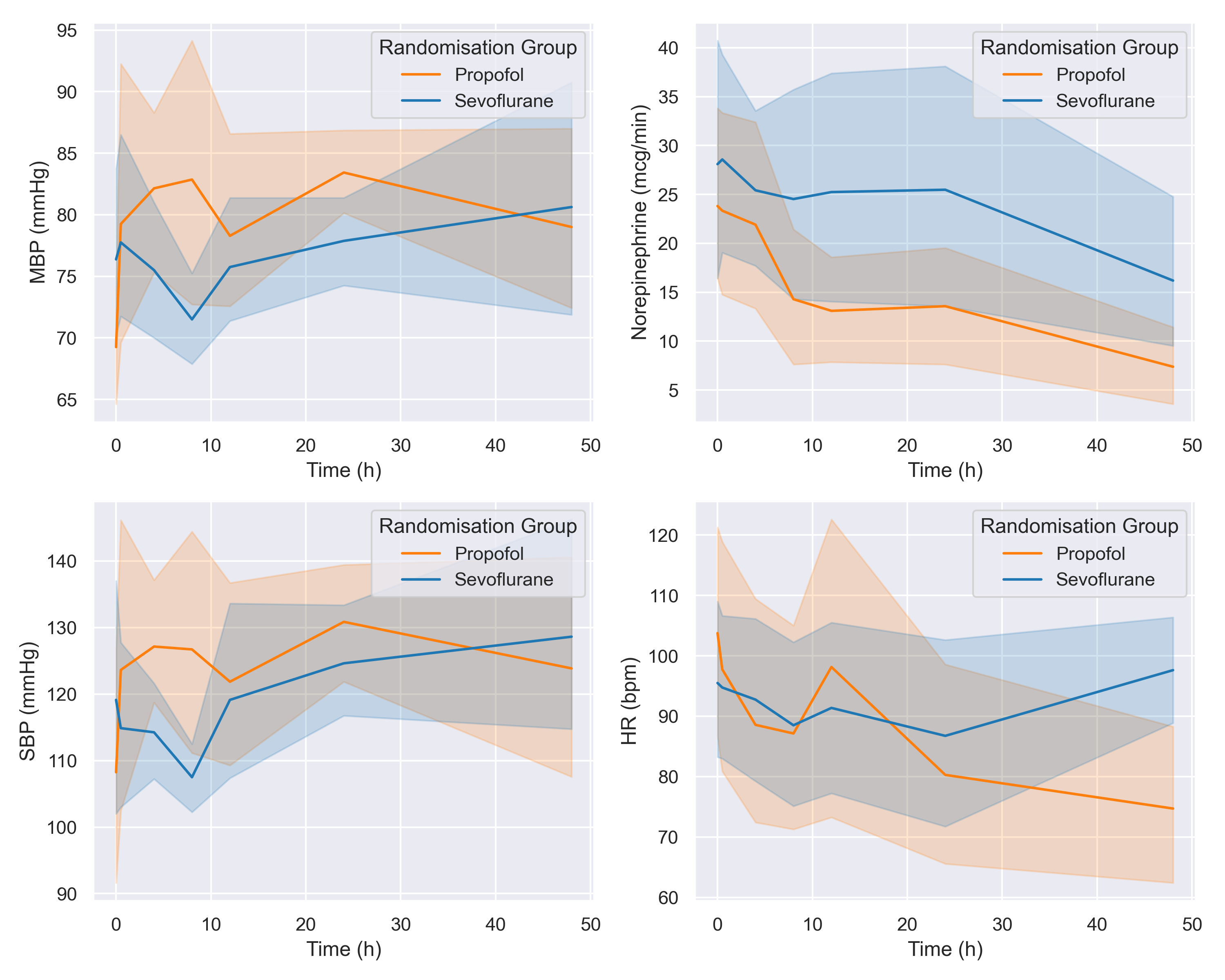

Supplement: Supplementary file 1 — Supplementary Material 1 [file 13613_2024_1311_MOESM1_ESM.tiff]

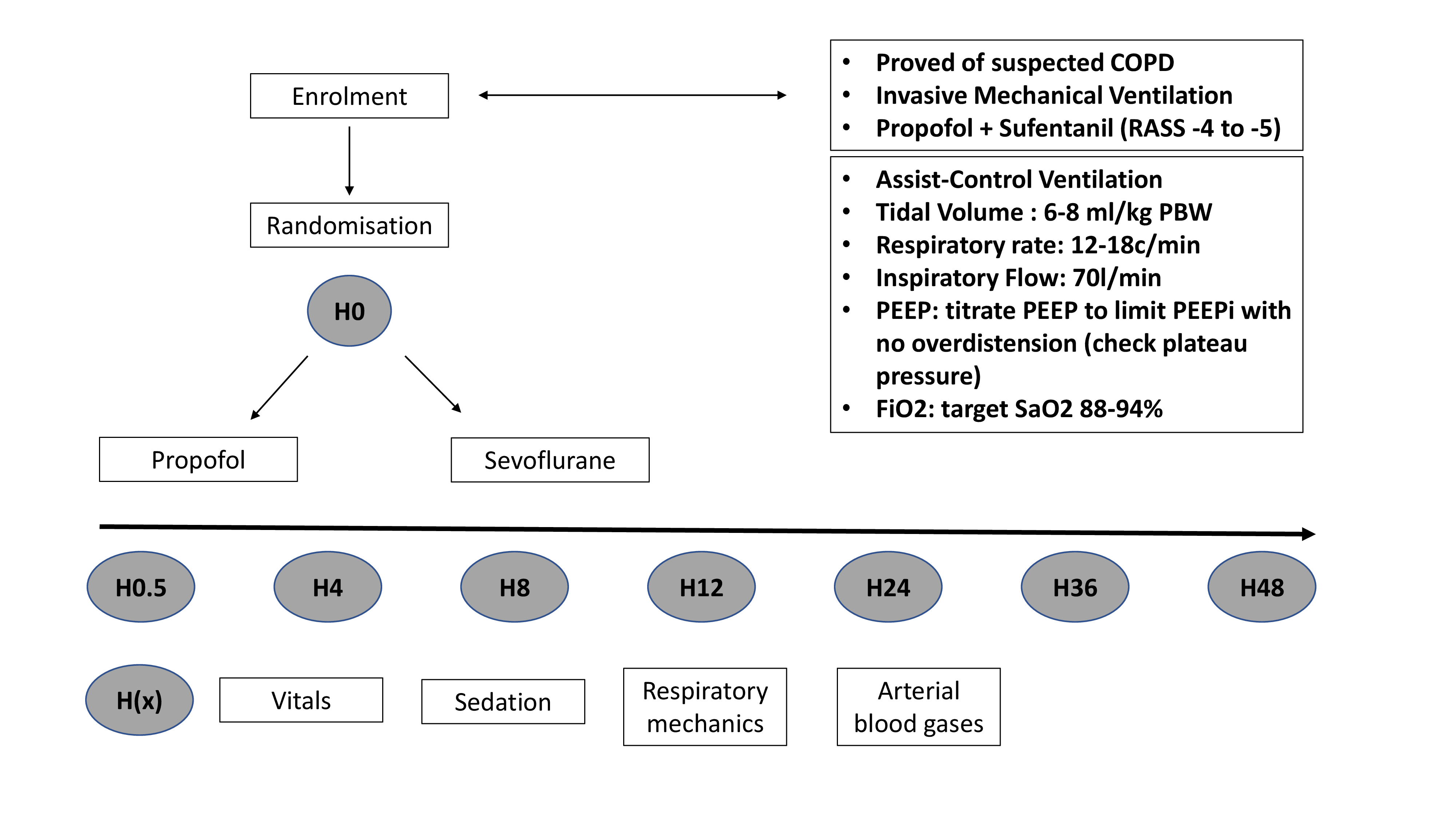

Supplement: Supplementary file 3 — Supplementary Material 3 [file 13613_2024_1311_MOESM3_ESM.tiff]
