# Supplementary material for: Early and late effects of volatile sedation with sevoflurane on respiratory mechanics of critically ill COPD patients
Source: Ann Intensive Care. 2024 Jun 18;14:91. doi: 10.1186/s13613-024-01311-4 (PMC11189368; doi:10.1186/s13613-024-01311-4)
Supplement: Supplementary file 2 — Supplementary Material 2 [file 13613_2024_1311_MOESM2_ESM.docx]

***Supplemental Material***

**Early and late effects of volatile sedation with sevoflurane on respiratory mechanics**

**of critically ill COPD patients**

Boris Jung ^a,b,d,e^, Maxime Fosset MD, MSc ^a,c^, Matthieu Amalric MD ^a^, Elias Baedorf-Kassis ^d,e^, Brian O’Gara MD ^d^, Todd Sarge MD ^d^, Valerie Moulaire, MD ^a^, Vincent Brunot, MD ^a^, Arnaud Bourdin, MD, PhD ^b,f^ , Nicolas Molinari PhD ^c^, Stefan Matecki MD, PhD ^b^

^a^: Medical Intensive Care Unit, Montpellier University and Montpellier University Health Care Center, 34295-Montpellier, France

^b^: PhyMedExp laboratory, Montpellier University, INSERM, CNRS, CHRU Montpellier, 34295-Montpellier, France

^c^: IMAG, CNRS, Montpellier University and Montpellier University Health Care Center, 34295-Montpellier, France

^d^ Department of Anesthesia, Critical Care and Pain Medicine, Beth Israel Deaconess Medical Center Harvard Medical School Boston, Massachusetts

^e^: Division of Pulmonary, Sleep and Critical Care Medicine, Beth Israel Deaconess Medical Center Harvard Medical School Boston, Massachusetts

^f^: Department of Respiratory Diseases, Montpellier University and Montpellier University Health Care Center, 34295-Montpellier, France

Corresponding author**:** Boris Jung, Medical Intensive Care Unit, Montpellier University and Montpellier University Health Care Center, 34295-Montpellier, France

e-mail: [b-jung@chu-montpellier.fr](mailto:b-jung@chu-montpellier.fr)

Key words**:** COPD, volatile sedation, sevoflurane, mechanical ventilation, respiratory mechanics

**E-Figure Legends**

**E-Figure 1:** Study design

**E-Figure 2:** Minute ventilation (ml), PaCO2 (mmHg) and PaO2/FiO2 (mmHg) displayed as median and quartiles from inclusion to H48 in the two groups. There was neither an early nor a late difference in total airway resistance between the two groups.

**E-Figure 3:** Mean blood pressure, systolic blood pressure, norepinephrine (mcg/min) administration and heart rate over time from inclusion to H48 in the two groups expressed as mean and SEM. There was no significant differences between the groups over time.
